# Obesity and Nutrigenetics Testing: New Insights

**DOI:** 10.3390/nu16050607

**Published:** 2024-02-23

**Authors:** Mychelle Kytchia Rodrigues Nunes Duarte, Lúcia Leite-Lais, Lucymara Fassarella Agnez-Lima, Bruna Leal Lima Maciel, Ana Heloneida de Araújo Morais

**Affiliations:** 1 Biochemistry and Molecular Biology Postgraduate Program, Biosciences Center, Federal University of Rio Grande do Norte, Natal 59078-970, RN, Brazil; mychellekytchia@hotmail.com (M.K.R.N.D.);; 2 Department of Cell Biology and Genetics, Biosciences Center, Federal University of Rio Grande do Norte, Natal 59078-970, RN, Brazil; lucia.leite@ufrn.br; 3 Department of Nutrition, Health Sciences Center, Federal University of Rio Grande do Norte, Natal 59078-970, RN, Brazil; bruna.maciel@ufrn.br; 4 Postgraduate Program in Nutrition, Federal University of Rio Grande do Norte, Natal 59078-970, RN, Brazil; 5 Postgraduate Program in Nutrition, Health Sciences Center, Federal University of Rio Grande do Norte, Natal 59078-970, RN, Brazil

**Keywords:** obesity, nutrigenetic testing, nutrition precision

## Abstract

Background: Obesity results from interactions between environmental factors, lifestyle, and genetics. In this scenario, nutritional genomics and nutrigenetic tests stand out, with the promise of helping patients avoid or treat obesity. This narrative review investigates whether nutrigenetic tests may help to prevent or treat obesity. Scientific studies in PubMed Science Direct were reviewed, focusing on using nutrigenetic tests in obesity. The work showed that few studies address the use of tools in obesity. However, most of the studies listed reported their beneficial effects in weight loss. Ethical conflicts were also discussed, as in most countries, there are no regulations to standardize these tools, and there needs to be more scientific knowledge for health professionals who interpret them. International Societies, such as the Academy of Nutrition and Dietetics and the Brazilian Association for the Study of Obesity and Metabolic Syndrome, do not recommend nutrigenetic tests to prevent or treat obesity, especially in isolation. Advancing nutrigenetics depends on strengthening three pillars: regulation between countries, scientific evidence with clinical validity, and professional training.

## 1. Introduction

Obesity is a complex chronic disease defined by excessive adiposity from exposure to obesogenic environments, psychosocial factors, and genetic variants [1]. It is a significant risk factor for the emergence of other chronic non-communicable diseases (NCDs), negatively impacting the well-being and life quality of those affected [2].

In 2019, this chronic condition contributed to approximately 5 million deaths from cardiovascular disease (CVD), diabetes, cancer, neurological disorders, chronic respiratory diseases, and digestive disorders [3]. If current trends continue, one billion adults (almost 20% of the world’s population) will be obese by 2025 [4].

Given this scenario, interventions are necessary and urgent. Many researchers try to delve deeper into understanding the complexity of the disease and which treatments would be most successful. At the 75th World Health Assembly in 2022, member states of the World Health Organization (WHO) adopted new recommendations for preventing and managing obesity, approving “The WHO Acceleration Plan to STOP Obesity”. This plan emphasizes that obesity is preventable by increasing the consumption of fruits, vegetables, and whole grains, limiting the intake of fats and sugar, and practicing regular physical activity. Furthermore, it highlights the importance of political efforts, encouraging self-care, and incorporating healthy eating and physical exercise [5].

In addition to diet and psychosocial factors, genetic factors can lead to the negative regulation of specific metabolic pathways and contribute to the onset of the disease [6]. Considering the genetic aspect, obesity can be monogenic or polygenic [4].

Monogenic obesity has a Mendelian inheritance pattern, involves genetic variations in a single gene or chromosomal region, has high penetrance, early onset, and is typically rare. On the other hand, polygenic obesity is more common than monogenic cases, results from hundreds of single nucleotide polymorphisms (SNPs) in several genes with low penetrance, and has a heritability pattern similar to other complex diseases [4].

Many genes associated with obesity are involved in regulating energy intake, lipid metabolism, adipogenesis, thermogenesis, adipokine synthesis, and transcription factors [7]. The genetic basis of polygenic obesity is diffuse, multifactorial, and non-deterministic. Many variants are distributed throughout the genome and have a small contribution to the onset of the disease, thus becoming a challenge for clinical practice. Information on existing genetic variants is necessary to characterize susceptibility to obesity [8].

Although challenging, offering personalized dietary advice based on an individual’s genetic susceptibility may be a promising strategy for preventing or treating obesity and diseases related to this chronic condition [9]. Thus, nutrigenetic tests can be an auxiliary tool in preventing and treating obesity, as they provide information on the primary SNPs, genotypes, possible predispositions to obesity, and directions for a better response to nutritional intervention [10].

Despite the existing concerns and challenges, there is great evidence for the potential of nutrigenetics in preventing and treating obesity and associated diseases [11,12,13]. This evidence points to promising perspectives regarding the use of nutrigenetic tests [14].

Considering the severity of obesity and the urgency of viable and resolute solutions for its management, nutrigenetics and precision nutrition have stood out as important prevention and treatment strategies. Thus, this narrative review provides a current overview of obesity, addressing in a conceptual, historical, and descriptive way the importance and real applicability of nutrigenetic tests in this context, highlighting their advantages and limitations.

## 2. Obesity: Epidemiology, Diagnosis, and Treatment

The WHO defines obesity as excess body fat resulting from a positive energy balance over time [15]. Globally, overweight and obesity affect more than 2 billion adults. By 2025, it is expected that 1 billion adults (more than 20% of the world’s population) will have obesity [4].

Excess adipose tissue not only influences the central regulation of energy homeostasis but can also become dysfunctional and predispose the individual to the development of comorbidities and complications [16], negatively impacting health and quality of life.

Both the excess and the ectopic location of body fat influence the production of adipokines and inflammatory mediators capable of altering glucose and lipid metabolism, leading to increased cardiometabolic and cancer risks and reducing life expectancy by 6 to 14 years [17]. It is estimated that 20% of all cancers can be attributed to obesity [18].

Obesity, as a complex multifactorial disease, is associated with an increased risk of developing several NCDs, such as CVD, 13 types of cancer, type 2 diabetes (DM2), and chronic respiratory diseases, including obstructive sleep apnea [19,20,21].

Cultural factors must also be taken into consideration in the etiology of obesity. For example, Asians tend to have a propensity for a lower body mass index (BMI) due to collectivistic cultures [22]. These cultures embrace cooperation and compliance with norms, unlike countries with a more individualistic culture, such as the United States (USA), where emotional and environmental triggers are the determining risk factors for weight gain [23]. A recent ecological analysis of 54 countries concluded that collectivism was significantly associated with a lower incidence of obesity in the population [24].

Diet is also a determining factor in the significant increase in obesity. Changes in the population’s dietary pattern, such as the increased consumption of ultra-processed products with low nutritional value and high levels of sodium, fats, or sugars, which have been replacing the consumption of fresh and minimally processed foods [25]. In several countries, a high consumption of ultra-processed foods has been associated with obesity due to an increased energy intake due to sugar consumption, decreased fiber consumption, and decreased protein density [26].

In Brazil, the national VIGITEL survey, a telephone survey to investigate and monitor risk factors for NCDs, detected a high consumption of ultra-processed foods by the population. Approximately 17.7% of Brazilians consume ultra-processed foods, 22.0% are men, and 14.1% are women. The consumption of these foods tended to decrease with age and was highest among schoolchildren aged 9 to 11 years old [27].

The global consumption of sweetened products and sugary drinks has grown in parallel with the obesity pandemic [28,29]. This direct association places sugar consumption as an essential risk factor for obesity [30,31]. Thus, the importance of food and nutritional education for the population, aiming to prevent diseases such as obesity, is highlighted.

Anthropometry is essential for diagnosing and assessing obesity, including body mass index (BMI), weight history, and body composition [32]. Using the BMI, it is possible to identify obesity (≥30 kg/m^2^), overweight (25–29.9 kg/m^2^), or eutrophy (18.5–24.9 kg/m^2^) [15]. For people with an increased BMI, waist circumference can identify increased visceral adiposity and cardiometabolic risk [16,33]. Furthermore, family history, clinical history, and biochemical data can make obesity treatment more individualized [32].

Many treatments are listed to eradicate obesity at individual and population levels. However, several have yet to succeed in the long term [34]. Behavioral and lifestyle interventions aimed at reducing energy intake and increasing energy expenditure have limited effectiveness because complex and persistent hormonal, metabolic, and neurochemical adaptations can hinder weight loss and promote weight regain [35,36].

In the USA, some medications are approved for the treatment of obesity, such as phentermine©, topiramat©, orlistat©, naltrexone©, bupropion©, liraglutide©, and semaglutide©. Often, these medications are prescribed in combination. In Europe, only orlistat©, naltrexone©, bupropion© and liraglutide© are approved [37]. Lorcaserin©, a selective serotonin 5C receptor (2-hydroxytryptamine) agonist, was recently withdrawn from the North American market due to concerns about increased cancer incidence in a cardiovascular outcome study [38]. In Brazil, three medications are approved for treating obesity: sibutramine©, orlistat©, and liraglutide© [39].

Sibutramine© blocks the reuptake of norepinephrine (NE) and serotonin (SE) and reduces food intake. Orlistat© is an analog of lipstatin, an inhibitor of gastrointestinal lipases, reducing the binding of approximately one-third of triglycerides and their absorption in the intestine. Liraglutide© is a glucagon-like peptide-1 (GLP-1) agonist, increasing the signaling of neurons synthesizing pro-opiomelanocortin and the transcript regulated by cocaine and amphetamine (*POMC*/*CART*). The medication indirectly inhibits neurotransmission in neurons that express neuropeptide Y (*NPY*) and agouti-related peptide (*AgRP*), favoring the weight loss process [40].

Endoscopic and surgical procedures also comprise an arsenal of strategies for controlling obesity. The intragastric balloon, duodenal mucosal resurfacing, and bariatric surgeries are among them. These procedures are indicated according to the degree of obesity, comorbidities, and the therapeutic effect sought [41,42].

Innovative therapeutic approaches are also researched for treating obesity and body weight regulation [37]. The new technologies of induced pluripotent stem cells (iPSC) and gene editing mediated by clustered, regularly interspaced short palindromic repeats are among them (CRISPR) [43].

Despite the various treatments available, combating obesity requires approaches that combine individual interventions with environmental and behavioral changes. Therefore, a better understanding of regional etiological differences in the prevalence of obesity can help identify the social causes of obesity and provide guidance on which intervention strategies are most promising [34]. Obesity is not caused by personal choice but rather by the relationship between an individual and their environment [34].

Thus, overweight and obesity result from an interaction between genetic and environmental factors. In this scenario, the emerging precision nutrition considers the main characteristics related to the individual (genotype, phenotype, diet, metabolic biomarkers, and intestinal microbiome) to establish personalized dietary recommendations that optimize the response to nutritional treatment. Therefore, precision nutrition is an essential complementary strategy for effectively treating obesity and its comorbidities [44].

## 3. Precision Nutrition and Exposome

In recent years, the concept of precision medicine or personalized medicine [45] became evident with a publication by Francis Collins [46], director of the National Institutes of Health (NIH). The director announced a new era and the creation of a national cohort aimed at recruiting and monitoring one million individuals to generate omics data [47].

Within precision medicine is precision nutrition. Precision nutrition aims to enable personalized dietary recommendations, optimize prevention, delay disease progression, and improve an individual’s health [9] through understanding the patient’s exposome.

In 2005, Wild developed the exposome concept, which comprises the totality of human exposures throughout life, from conception to the end of life [48]. This designation was improved and defined as the cumulative measure of environmental influences and associated biological responses throughout existence, including environmental exposures, diet, behavior, and endogenous processes [49].

Thus, an individual’s exposome must be considered, as well as a series of omics markers [47]. Precision nutrition must consider metabolic phenotyping using high-throughput omics technologies, such as genomics (polymorphisms and other structural genetic variants), epigenomics (DNA methylation, histone modifications, long non-coding RNA, telomere length), transcriptomics (patterns of RNA expression), proteomics (protein signatures), metabolomics (metabolite profiles), and metagenomics (intestinal microbiota composition, enterotypes), under a holistic approach comprising nutritional genomics [50].

## 4. Nutritional Genomics

With the Human Genome Project, nutritional genomics emerged as a field of research to assist in diagnosing, preventing, and managing chronic diseases influenced by diet [51]. Nutritional genomics studies how genes and nutrients interact and influence phenotypes, including disease risk [52]. Nutrigenomics, nutrigenetics, and nutritional epigenomics are subareas of nutritional genomics, and each studies different aspects. Nutrigenomics is a part of omics sciences that studies the influence of nutrients and diet on genes, proteins, and metabolites. Nutrigenetics studies the impact of genetic variations, mainly SNPs, on individual responses to nutrients and diet. These genetic variations can influence protein synthesis and functions, thus modifying dietary needs and metabolism, and may impact the development of diseases. Nutritional epigenomics investigates the impact of nutrients and diet on changes in the human genome that do not involve changes to the DNA sequence but affect gene expression, extending from gene activation to protein synthesis [53].

As nutritional genomics promises to transform global health and medicine, there is growing interest in the relationship between genotype and phenotype. The phenotype derives from genetic and environmental contributions [54]. Once “the” genetic variants that may predispose a trait or disease have been identified, the next challenge is to decode the genetic variation that explains heritability, in addition to the epigenetic changes [55]. Therefore, it is essential to know the genetic profile of obesity to identify how many variants are involved in this chronic condition and direct the use of nutritional genomics in patients’ treatment.

## 5. Genetic Aspects of Obesity

Obesity is genetically classified into monogenic or polygenic. Monogenic obesity is a rare, severe, early-onset form with a Mendelian inheritance pattern, high penetrance, and significant genetic effect [4]. In contrast, polygenic obesity is more prevalent. It has a heritability pattern derived from many variants in several genes with low penetrance [56].

**Figure 1:**
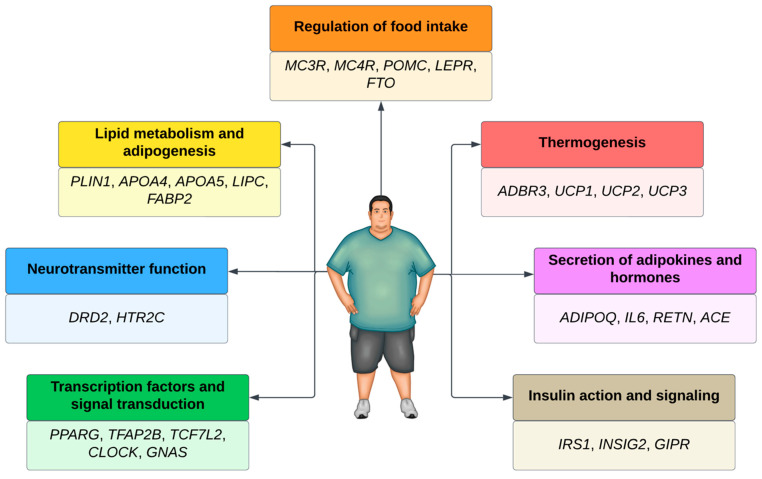
Main genes and pathways associated with obesity.

Several genes associated with polygenic obesity have been found in Genome-Wide Association Studies (GWAS). Most of these genes are involved in the leptin–melanocortin pathway, which regulates food intake [56]. Other pathways are also involved in the development of polygenic obesity (Figure 1).

Over 1100 obesity-associated loci have already been identified in approximately 60 GWAS [4]. Almost all chromosomes in the human genome (except the Y) contain at least one locus associated with body weight regulation [57]. When considering those that are BMI- and obesity-associated, more than 250 loci were identified [58].

Scientific evidence shows that, despite the discovery of many genetic loci susceptible to obesity, the size of each variant’s effect on BMI is small [59], possibly due to the influence of environmental factors. Therefore, some environmental factors, such as physical activity, diet, and smoking, have been considered in the analyses of some GWAS. However, this approach is challenging as determining the effects of these gene–environment interactions on new biological insights is a complex task. It is estimated that only 12 loci associated with obesity and influenced by environmental factors have been identified [4].

A meta-analysis identified nine loci with convincing evidence of an interaction between smoking as an environmental factor and genes associated with obesity, as evidenced by BMI and waist circumference. Thus, smoking can alter genetic susceptibility to general adiposity and body fat distribution [60].

In genes associated with obesity, the presence of thousands of genetic variants can influence the etiology of obesity. The most studied genetic variants are single nucleotide polymorphisms (SNPs), represented by a single nucleotide change concerning the reference sequence at a specific position in the genome. Scientists have found more than 600 million SNPs in human populations worldwide [61]. These minor genetic variations determine phenotypic differences between individuals [62]. Patients with SNPs in pro-opiomelanocortin (*POMC*) and the melanocortin 4 receptor (*MC4R*) are more prone to excessive body fat accumulation. Individuals with SNPs in the fat mass and obesity gene (*FTO*) [63] and the dopamine 2 receptor (*DRD2*) are more predisposed to binge eating.

SNPs in beta-adrenergic receptors 3 (*ADRB3*) and perilipin (*PLIN*) increase the predisposition to adipogenesis or lipid metabolism imbalances. SNPs in the uncoupling protein gene (*UCP*) lead to difficulty in energy expenditure, while SNPs in insulin receptors (ISR-2), adiponectin (*ADIPOQ*), and interleukin 6 (*IL-6*) [64] predispose patients to more significant oxidative stress in the body and make weight loss difficult. Thus, all these SNPs can influence the emergence of obesity. Genetic tests targeting SNPs have become accessible to the population, allowing genetic information to direct therapeutic strategies [65].

## 6. Genetic Testing (GT)

Genetic tests (GT) are those that, from a biological sample, can directly examine the DNA or RNA that constitutes a gene (direct test), observe markers inherited together with a disease-causing gene (linkage test), examine the protein products of genes (biochemical test), or examine the entire chromosome (cytogenetic test) [66].

There are several types of GT, each with a different purpose (e.g., newborn screening, carrier testing, prenatal diagnostic testing, genetic tests, predictive genetic testing, and forensic testing) [67]. Among them, diagnostic GT is used to confirm the disease of a symptomatic individual. On the other hand, predictive GT identifies genetic variations that increase a person’s risk of developing a particular disease or clinical condition. The clinical usefulness of GT depends on the evidence obtained about how much the genetic variant can contribute to the diagnosis, prognosis, or management of the disease [68].

GT generally raise concerns from consumers, healthcare professionals, and regulators. These concerns are not new and affect the individual and public health. They address ethical, legal, psychological, and clinical issues. Autonomy, confidentiality, privacy, and equity are ethical and legal aspects related to the commercialization and use of these tests, and the storage of genetic information [69].

As for the psychological impact, these tests can generate fear of discrimination, anxiety, and depression, depending on the client’s perception regarding the risk, severity, and the possibility of treatment for the diseases listed [68]. Clinical concerns are inherent to the results’ robustness and applicability [70]. Therefore, some authors agree to limit access to TG for specific clinical conditions (e.g., Alzheimer’s disease) until truly effective treatments exist [71]. However, considering the substantial benefits of genomics for personal and public health, the WHO supports disseminating and implementing genomic technologies, including GT, with ethical and legal responsibility [72].

GT regulation varies significantly between countries. Despite existing international regulations, each country has the autonomy to modify and apply them. Technological advances, medical utility, access, and societal acceptance have contributed to more permissive regulations and greater use of GT [73]. Although genetic counseling is encouraged, few countries legally require it or recognize it as a profession [74].

In the United States, federal regulations evaluate and regulate GT based on three criteria: analytical validity, clinical validity, and clinical utility. Analytical validity concerns the accuracy of the test in detecting whether a genetic variant is present. Clinical validity indicates evidence of the relationship between a genetic variant and the presence, absence, or risk of a particular disease or clinical condition. Clinical utility matches the test’s ability to contribute to better health outcomes [75,76]. The WHO discusses ethical, legal, social, and regulatory issues related to the use of genomic methods and affirms the importance of the global development of rules, technical standards, and sensible policies so that access to and ownership of data is assertive for those who use it and can use it while benefiting, or not, from the use of genomic information [15].

### 6.1. Direct-to-Consumer Genetic Testings (DTC-GT)

After completing the human genome project in 2003, research further advanced the investigation of genetic variations, especially SNPs, and disease risks. This fact, associated with the ease of microarray technology and GWAS, boosted the emergence and commercialization of direct-to-consumer genetic testing (DTC-GT) without needing a prescription or medical referral [77]. DTC-GT is also called “over-the-counter genetic testing”, “at-home genetic testing”, or “home DNA testing” [78]. These are not diagnostic tests but predictive and pre-symptomatic tests that assess the risk or genetic susceptibility to certain diseases or clinical conditions, such as Alzheimer’s disease, cancer, diabetes, and CVD [70]. Thus, DTC-GT allows people to access and understand their genetic information without necessarily involving a healthcare provider [78].

Predictive DTC-GT can generate uncertain results regarding the development of a clinical condition. However, it has a beneficial potential for screening, surveillance, and prevention strategies that can reduce morbidity and mortality [79,80]. They even have different clinical utility hierarchies between diseases [79]. Over the years, prior uncertainties, misunderstandings, and caution regarding predictive GT [70,81] have gradually been replaced by increased acceptance among populations in several countries [80,82,83,84,85,86].

To carry out DTC-GT, consumers purchase test kits online (most commonly), in stores [78], and by email or telephone [65]. With step-by-step instructions, consumers collect their biological sample (usually saliva), send their data by mail to the company, and, after laboratory analysis, receive their results by email or on an online platform upon account registration [78,87].

Some companies collect additional customer data and request terms of service agreements to be signed [88]. Supposing the consumer wishes to have their information used in research, a separate consent form must be signed for the company to share data with third-party collaborators [89]. However, consent forms can have a problematic reading level for a layperson, or companies can induce consent by an easy click required to finalize the order [90]. As results may be incomplete or complex to interpret [89], some companies deliver more detailed reports [91] and often offer tailored diets, nutrition supplements, meals, and exercise plans [87,92].

### 6.2. Nutrigenetic Tests

DTC-GT offer several services; the most common are related to ancestry, disease risks, and lifestyle, including physical activity and diet [91,93]. These tests have impacted the personalization of pharmacotherapy (pharmacogenetics) [77] and diet therapy (nutrigenetics) [73]. Nutrigenetics tests are DTC-GT focused on nutrigenetics as they evaluate genetic variations (e.g., SNPs) related to monogenic or polygenic changes and contribute to more personalized nutritional guidance. For example, using specific genotypes, it is possible to verify predisposition or susceptibility to intolerance and sensitivity to food compounds (e.g., caffeine, lactose, gluten), changes in energy and nutrient metabolism (e.g., fatty acids, folate), obesity, and dietary needs for specific vitamins and minerals [91].

Nutrigenetic tests were one of the first types of DTC-GT offered [87], and since then, they have been commercialized by several companies in several countries around the world. Philip et al. (2016) mentioned that 72 companies offered the nutrigenetic testing service, representing 30% of the categories offered by the 246 companies that provided DTC-GT [92]. According to Floris et al. (2020), 45 companies worldwide sell nutrigenetic tests [65]. Their largest concentration is in Europe (n = 21) and North America (n = 19), four of which are multinationals.

The main requirement of a nutrigenetic test is to specify a dietary recommendation that is proven beneficial to the individual. The results must be consistent and have compelling evidence in replicated studies to achieve this [94]. However, in cases of polygenic conditions or diseases, where there is a high influence between genetic and environmental factors [91], many genetic variations analyzed in nutrigenetic tests still provide inconclusive or unreliable information, limiting personalized dietary recommendations, and disagreeing on ethical issues [94]. In line with this, a recent review highlights that the lack of knowledge, skills, and evidence-based information are the main factors limiting the use of nutrigenetics in clinical practice [14]. On the other hand, these limitations are seen as opportunities for improvement [95].

Despite these limitations, people have great interest and positive attitudes towards nutrigenetic tests. A European multicenter study investigated the opinions of almost 6000 participants about nutrigenetic tests. Among the participants, 66% were willing to carry out this type of test, and 27% would like to follow a personalized diet. Most of these people had chronic conditions such as dyslipidemia, central obesity, and high levels of stress [96].

Another study in Quebec investigated the attitudes, perceptions, and concerns of 1425 individuals about nutrigenetic tests. The main advantages reported by participants regarding using nutrigenetic tests were health and disease prevention. Dietary restriction was the main disadvantage reported, but was only pointed out by a minority. The biggest concerns were access to and use of personal genetic information [10]. Interestingly, studies demonstrate that nutrigenetic tests help motivate people to adhere to the diet and incorporate a healthy lifestyle [91,97], contributing to the control of weight [14] and proactivity about health [78].

Although nutritionists are considered the best professionals to provide personalized dietary advice based on nutrigenetic testing [10], many still need to be qualified, and a higher level of education/training is necessary [98]. For this reason, and given the need for more significant scientific evidence, position statements issued by associations state that caution must be taken when interpreting and using information from the nutrigenetic tests [52,62,99]. Although individuals respond differently to a given diet, nutrient, or bioactive compound due to their genetic variations, nutrigenetic tests can assist in nutritional intervention/guidance. However, they should never be used in isolation to personalize the diet [62]. Despite the mechanisms of interactions between genes and diet that have already been revealed, scientific evidence that supports personalized nutritional recommendations through nutrigenetic tests is still scarce [94]. There is a need for more randomized clinical trials [99] to understand the targets of nutrigenetics testing.

### 6.3. Targets of Nutrigenetic Tests

Nutrigenetic recommendations can be classified and grouped into portfolios. These dietary recommendations are based on scientific evidence representing a basis for creating nutrigenetic standards established by different populations’ genetic and physiological characteristics worldwide [100].

The characteristics most investigated in predictive nutrigenetic tests belong to the “micronutrients” category. In 2020, thirteen companies provided DNA testing for genetic variants associated with vitamin D metabolism, ten for vitamin C, and nine for vitamins B12, A, and B6. Among minerals, iron metabolism is the most tested by four companies. Regarding macronutrient metabolism, lipid metabolism testing is provided by thirteen companies; carbohydrate metabolism and protein metabolism are tested by six companies and one company, respectively. Notably, lactose intolerance and caffeine metabolism tests are the most requested [65].

Out of 45 companies identified, only 16 declared the genes or genetic variants used in nutrigenetic predictions. Furthermore, only 50% of companies specified the dbSNP of the variants. This fact makes interpreting reports and assessing scientific reliability complex [65].

Powerful statistical tools have been created to assess the risk of having a specific phenotype, for example, the polygenic risk scores (PRS). The PRS combine multiple associated genetic variants into a single score, weighting their frequency in the population and their estimated impact on a given characteristic [101]. The PRS derived from the cumulative effect of genetic variants associated with obesity can help estimate the risk of developing this condition [102]. Thus, the PRS represent a promising advance in understanding the genetic risk of obesity [103].

In addition to the PRS, the polygenic score (PGS) is currently being discussed. Despite being considered synonymous with PRS by some, it is a more comprehensive term that includes rare or common variants and can be used when risk analysis does not apply. Furthermore, the PGS has the potential to expand understanding of the scope and role of DTC-GT [104].

### 6.4. Evidence from Nutrigenetics Testing in Obesity

Nutrigenetic recommendations are possible tools to complement standard dietary recommendations for preventing and controlling obesity and its comorbidities [13]. Nutrigenetic recommendations address individualized nutritional needs, considering genetic characteristics [105,106,107]. Studies have demonstrated the potential of using nutrigenetic tests to manage obesity. However, few studies describe the evidence of nutrigenetic tests marketed as DTC-GT in this context (Table 1).

Even with few scientific studies evaluating the impact of nutrigenetic tests, such as DTC-GT, in clinical practice, there is a significant increase in commercializing these tests, whether through spontaneous demand or via professionals in private assistance [14]. Despite the positive evidence demonstrated in weight loss and weight maintenance (Table 1), more research is needed to scientifically validate commercialized nutrigenetic tests, as confirmed by some studies [105,107,108]

A systematic review aiming to evaluate the use of genetic information in dietary advice showed that incorporating GT did not significantly improve or worsen food intake compared to the control group. However, the authors emphasize that these results should be interpreted cautiously due to the limited number of studies available, heterogeneity in design, and selected genetic markers [110]. The systematic review confirms that clinical trials need to be better designed to justify the use of GT in nutritional counseling. The authors also comment that the pediatric population was outside the scope of the work, highlighting the paradigm of clinical practice complexity, which must involve genetic information, microbiome data, and omics sciences for better treatment [110].

Li and colleagues reviewed 13 studies and found that genetic counseling did not improve motivation to change dietary intake [111]. Another review evaluating 18 studies found that disclosing genetic information did not alter eating behavior [112].

Some professional associations, such as the Brazilian Association for the Study of Obesity and Metabolic Syndrome (ABESO) [32] and the Academy of Nutrition and Dietetics [52] do not endorse the use of dietary recommendations based on nutrigenetics testing in clinical practice.

Furthermore, continued education strategies must be aimed at health professionals, who need to interpret these tests better. Ethical issues concern the scientific community and nutrigenetic test consumers [14], which need more discussion and regulation.

### 6.5. Overview of Ethical Issues and Positions on Nutrigenetics Tests

Regarding the practical and ethical barriers related to nutrigenetics tests, Bates et al. (2005) point out that the information provided by the tests still needs to be discussed [113]. Qualitative studies in the United Kingdom, still considering the controversies surrounding the UK Biobank project, reinforced distrust in the government’s ability to regulate the use of genetic information [81].

In the case of the USA, in the early 2000s, concerns involved the potential misuse of samples, a lack of confidentiality, misuse of information by insurers and employers, discrimination based on genotype, and commercial exploitation of information and technology [114,115]. Belgium did not have specific legislation regarding GT then, and non-binding guidance documents still governed the rules and standards. In Belgium, there is no distinction between diagnostic and predictive tests; however, experienced physicians generally order predictive tests [116].

German legislation has established that a doctor can only perform a GT after providing sufficient information and appropriate genetic counseling [117]. In Italy, there are more general authorizations and guidelines depending on the type of test [97].

In China, the Ministry of Health has attempted to oversee genetic counseling by developing guidelines for clinical genetic counseling. Furthermore, China recently announced that it is developing a guide for consumers to make informed decisions about nutrigenetic tests and for companies to provide information and messages that are not misleading to the consumer [118].

There are few legislative controls regulating GT use in Canada, and the situation worsens for nutrigenetics tests. In 2017, the Parliament of Canada approved Bill S-201, the Genetic Non-Discrimination Act (GNDA), which prohibited and prevented genetic discrimination [119]. This new law aimed to protect consumers from discrimination by employers based on the results of a GT, eliminate the requirement for an individual to undergo GT, and prohibit insurance companies from requiring GT results [14]. In December 2018, the Quebec Court of Appeal considered that Sections 4 to 9 of the GNDA were ‘ultra vires’, meaning that these sections were unconstitutional and, as a consequence, the GNDA is not a valid law, and the legitimacy of the GNDA will be determined by the Supreme Court of Canada [14].

In addition to controversial ethical issues, there is the position of Academies and Societies regarding nutrigenetic tests. The Academy of Nutrition and Dietetics (USA) and the Brazilian Society of Food and Nutrition (Brazil) do not recommend the isolated use of nutrigenetic tests for the development of dietary plans and the prescription of dietary supplements [52,62,99]. ABESO also does not recommend using isolated nutrigenetic tests to treat obesity due to insufficient scientific evidence [120]. Therefore, more research should be carried out attempting to use nutrigenetic tests in each country and evaluate their use in the prevention and treatment of diseases.

## 7. Conclusions

Despite evidence for the benefits of nutrigenetics and nutrigenetic testing in the prevention and treatment of obesity, there are still significant challenges and ethical issues debated by regulatory and professional institutions. However, a shared conclusion is that nutrigenetic tests should not be used in isolation in the treatment of obesity but can provide important information in individual obesity management or at a public health level. It is necessary to strengthen three pillars to advance nutrigenetics: regulation, evidence, and education. Countries need to communicate with each other and evolve in their regulations on GT and the use of genetic information, including those related to nutrigenetics. More well-designed nutrigenetics studies with robust methodologies are necessary to strengthen nutrigenetics tests’ scientific evidence and clinical validity. Finally, it is essential to educate and prepare health professionals, especially nutritionists, regarding nutrigenetics so that they can safely recommend the nutrigenetic tests, interpret their results, and outline more personalized and effective diet therapy approaches for people with predispositions to or who are diagnosed with obesity.

## Figures and Tables

**Table 1:** Scientific evidence of nutrigenetic tests in obesity.

Author (Year)	Country	Nutrigenetic Company and the Number of SNPs Tested	Study Type	Aim of the Study	Participants	Main Results
Tamilvanan, Kalpana (2023) [107]	India	Nutrigenetic test (not specified); 15 SNPs tested in 10 genes involved in body weight and metabolism	Observational	To examine the usefulness of nutrigenetic testing in designing personalized diets and its potential to enhance weight loss	Healthy adults (n = 106) with BMI 25–40 kg/m^2^ and previous failures of weight loss maintenance. Nutrigenetic group (n = 54) and non-nutrigenetic group (n = 52)	The nutrigenetic group significantly reduced waist circumference and BMI at 60 and 90 days follow-up than the non-nutrigenetic group.
Vranceanu et al. (2020) [108]	Romania	NutriGENE by Eurogenetica Ltd./DNAfit, London, UK. Tested 28 SNPs in 22 genes with evidence of gene-diet/lifestyle interactions	Observational	To observe weight loss and biochemical parameters of participants following two different diet plans: ketogenic diet or low-glycemic index nutrigenetic (low-GI/NG) diet	Overweight and obese subjects (n = 114). Keto group (n = 53) and low-GI/NG group (n = 61)	After 24 weeks, the keto group lost more weight. However, at the 18-month follow-up, the low-GI/NG group lost significantly more weight and had more significant improvement in total cholesterol, HDL-c, and fasting glucose.
Frankwich et al. (2015) [109]	United States	Pathway Genomics, Inc., San Diego, CA, USA. Tested seven SNPs in seven genes involved in body weight and metabolism	RCT	To evaluate whether participants who followed a nutrigenetic-guided diet lost ≥ 5% of their body weight than participants on a standard balanced diet for 8 and 24 weeks	Obese or overweight US veterans (n = 51) were randomly assigned to groups placed on a nutrigenetic-guided diet (n = 30) or a standard balanced diet (n = 21)	No significant differences regarding weight loss, BMI, and waist circumference, among other outcomes, were observed between the groups.
Arkadianos et al. (2007) [105]	Greece	Sciona MyCellf kit (Sciona Inc., Boulder, CO, USA). Tested 24 SNPs in 19 genes involved in metabolism.	Clinical trial	Evaluate whether the use of nutrigenetic testing could promote long-term weight management.	Patients with a history of unsuccessful attempts at weight loss. Nutrigenetic group (n = 50) and control group (n = 43)	After 300 days, the nutrigenetic group had better long-term BMI reduction and improved blood fasting glucose.

SNP—single nucleotide polymorphism; BMI—body mass index; RCT—randomized controlled trial; US—United States.
